# Syndrome de Rhupus: à propos de deux observations

**Published:** 2012-06-26

**Authors:** Imen Hachicha, Hela Fourati, Rim Akrout, Sofien Baklouti

**Affiliations:** 1 Service de Rhumatologie CHU Hédi Chaker, 3029 Sfax, Tunisie

**Keywords:** Polyarthrite rhumatoïde, Lupus érythémateux systémique, Rhupus

## Abstract

L'association lupus érythémateux systémique et polyarthrite rhumatoïde (rhupus)est une condition clinique rare. A travers le monde 50 cas de Rhupus ont été décrits. Nous en rapportons deux nouvelles observations de patientes Tunisiennes qui présentaient une polyarthrite érosive à prédominance distale avec des anticorps anti Sm positifs dans un cas et des anti-DNA natifs dans l'autre cas.

## Introduction

La coexistence de deux ou plusieurs connectivites chez un même patient est un phénomène rare, en particulier la coexistence de polyarthrite rhumatoïde (PR) et de lupus érythémateux systémique (LES). Elle est décrite pour la première fois par Shur en 1971 sous le terme de Rhupus [1]. Toone [2] avait rapporté la première observation clinique en 1960 pour une meilleure identification de cette entité. A travers le monde, 50 cas de rhupus ont été décrits [3]. Nous en rapportons deux nouveaux cas.

## Patients et observation

### Observation 1

Patiente M.H. âgée de 45 ans sans antécédents pathologiques particuliers hospitalisée en Mai 2006 pour polyarthrite chronique touchant les deux poignets et les deux chevilles évoluant depuis une dizaine d'années non explorées, en association avec un érythème de visage d'apparition plus récente depuis 1 mois qui s'accentue à l'exposition solaire. L'interrogatoire ne trouvait pas de notion d'amaigrissement ni de chute de cheveux ni de xérostomie ou xérophtalmie. L'examen trouvait une patiente en bon état général, apyrétique, un érythème du visage en vespertilio, une synovite bilatérale des deux poignets sans déformation au niveau des mains. A la biologie, la numération formule sanguine a révélé une lymphopénie à 1200 éléments par mm^3^, la vitesse de sédimentation était à 30 mm à la première heure. Le bilan rénal ne trouvait pas d'anomalie du sédiment urinaire. La recherche des facteurs rhumatoïdes (FR) par la méthode ELISA était positive à 65 UI/ml. Les anticorps anti-peptides citrullinés (anti-CCP) étaient positifs à 122 RU/ml et les anticorps antinucléaires à 1/160 de type homogène avec des anti Sm positifs. La radiographie des mains montrait la présence d'une carpite stade III à droite et II à gauche. La radiographie des avant pieds trouvait une déminéralisation en bandes des têtes métatarsiennes. Le diagnostic de rhupus a été retenu devant la présence de 4 critères de l'ACR pour la PR et 4 critères de l'ARA pour le LES. La patiente a été traitée par corticothérapie à la dose de 10 mg/j en association avec des antipaludéens de synthèse.

### Observation 2

Patiente GN, âgée de 34 ans suivie pour polyarthrite rhumatoïde érosive et séropositive depuis 2001, le diagnostic est retenu selon les critères de l'ACR 1987. La patiente est traitée par Methotrexate à la dose de 15 mg par semaine en association avec la salazopyrine à la dose de 2g par jour. Elle a été hospitalisée en 2007 pour poussée de polyarthrite touchant les 2 poignets, les 2 coudes et les 2 genoux. Par ailleurs, on trouvait à l'interrogatoire la notion de xérostomie, xérophtalmie et de chute de cheveux dans un contexte d'amaigrissement et d'altération de l’état général. L'examen trouvait une patiente apyrétique, cachectique, une synovite bilatérale des deux poignets, une synovite des deux coudes avec un flessum à 15° bilatéral sans nodules rhumatoïdes palpables et une arthrite des deux genoux. L'examen ophtalmologique a objectivé un syndrome sec oculaire, et l'examen anatomopathologique de la biopsie des glandes salivaires a trouvé un grade III de Chisholm. A la biologie, la numération formule sanguine a révélé une anémie inflammatoire avec une hémoglobine à 10,6 g / dl, une leuco lymphopénie avec des globules blancs à 2700 éléments par mm^3^ et des lymphocytes à 500 éléments par mm^3^. La vitesse de sédimentation était à 110 mm à la première heure et une C-réactive protéine à 13 mg /l. Le bilan rénal a montré une leucocyturie sans germe à 332 000 leucocytes par minutes. La recherche des facteurs rhumatoïdes (FR) par la méthode ELISA était positive à 78 UI/ml. Les anticorps anti-peptides citrullinés (anti-CCP) étaient positifs à 100 RU/ml et les anticorps antinucléaires à 1/1280 avec des anti DNA et des anti SSA positifs. La radiographie des mains a montré la présence d'une carpite stade III bilatérale et la radiographie des avants pieds une arthrite érosive des métatarsophalangiennes. Le diagnostic de rhupus a été retenu devant la présence de 4 critères de l'ACR pour la PR et 4 critères de l'ARA pour le LES. La patiente a été traitée par corticothérapie à forte dose 1mg/Kg/j devant l'atteinte hématologique et l'atteinte rénale en association avec le Methotrexate et la salazopyrine. L’évolution était marquée par l'amélioration aussi bien sur le plan clinique que biologique.

## Discussion

Le rhupus est une entité clinique rare [4, 5]. Sa prévalence est de l'ordre de 0,09 % [4]. La revue de la littérature trouve plutôt des cas isolés. Une seule série Mexicaine a rapporté un nombre élevé de 22 cas de rhupus [3]. Les principaux diagnostics différentiels sont la PR avec des atteintes extra articulaires, LES avec polyarthrite ou bien une connectivite mixte. Le diagnostic de rhupus est alors porté quand l'une de ces pathologies ne valident leurs critères diagnostiques. Certains auteurs considèrent que le rhupus est un état de chevauchement entre PR et LES puisque les anti-CCP qui sont hautement spécifiques de la PR et les anti DNA/Sm qui sont hautement spécifiques du LES existent tous les deux dans le rhupus [6].

Les critères diagnostiques du rhupus selon certains auteurs sont: la présence de polyarthrite symétrique érosive, de manifestations cliniques de LES et la présence d'anticorps anti DNA ou anti Sm [3, 7].

Son étiopathogénie est un sujet de controverse et fait inclure des facteurs génétiques avec une fréquence élevée des allèles HLA DR1/ HLA RD2, des facteurs hormonaux surtout les hormones sexuelles et des facteurs environnementaux [3, 8–10]. Sur le plan clinique, les patients sont le plus souvent déjà suivis pour PR puis développent secondairement des signes de LES. Parfois, ils se présentent pour des signes de PR et de LES simultanés. Rarement des signes de LES sont au premier plan. Les symptômes classiques de la PR sont prédominants et précédent ceux du LES dans le temps [3, 4]. Particulièrement, les nodules rhumatoïdes sont fréquents et se voient dans 40 % des cas [3, 4].

Les manifestations cliniques du LES qui se voient fréquemment dans le rhupus sont l'atteinte cutanée avec photosensibilité, l’érythème du visage, l'alopécie; l'atteinte hématologique avec leucopénie et thrombopénie et la sérite avec pleurésie ou péricardite. Les atteintes neurologique et rénale sont rapportées plus rares [3, 4, 11, 12]. A la biologie, la prévalence des anti-CCP au cours de la PR (86 %) est significativement supérieure à leur prévalence au cours du rhupus (57 %) qui est significativement supérieure à leur prévalence au cours du LES (4,5 %) [6, 13]. Une fréquence élevée des anticorps anticardiolipines a été rapportée, cependant le risque thromboembolique paraît faible [3]. Sur le plan thérapeutique, il n'y a pas de consensus. Cependant, ces cas de rhupus doivent être reconnus car le pronostic peut être différent d'un LES seul ou d'une PR isolée [14].

## Conclusion

L'association PR-LES est une entité clinique rare et reste un diagnostic d’élimination. L’étiopathogénie est un sujet de controverse. Les signes cliniques de la PR sont prédominants. Le syndrome des antiphospholipides est rare malgré une fréquence élevée des anticorps anticardiolipines. La prise en charge thérapeutique pose souvent des problèmes.
